# Ginseng for Health Care: A Systematic Review of Randomized Controlled Trials in Korean Literature

**DOI:** 10.1371/journal.pone.0059978

**Published:** 2013-04-01

**Authors:** Jiae Choi, Tae-Hun Kim, Tae-Young Choi, Myeong Soo Lee

**Affiliations:** 1 Medical Research Division, Korea Institute of Oriental Medicine, Daejeon, Republic of Korea; 2 Department of Spine Center, Mokhuri Neck & Back Hospital, Seoul, Republic of Korea; University of Strathclyde, United Kingdom

## Abstract

**Objective:**

This systematic review was performed to summarise randomised clinical trials (RCTs) assessing the efficacy and safety of ginseng in the Korean literature.

**Method:**

The study involved systematic searches conducted in eight Korean Medical databases. The methodological quality of all of the included studies was assessed using the Cochrane Risk of Bias tool. We included all RCTs on any type of ginseng compared to placebo, active treatment or no treatment in healthy individuals or patients regardless of conditions.

**Results:**

In total, 1415 potentially relevant studies were identified, and 30 randomised clinical trials were included. Nine RCTs assessed the effects of ginseng on exercise capacity, cognitive performance, somatic symptoms, quality of life, and sleeping in healthy persons. Six RCTs tested ginseng compared with placebo for erectile dysfunction, while another four studies evaluated the effects of ginseng against no treatment for gastric and colon cancer. Two RCTs compared the effect of red ginseng on diabetes mellitus with no treatment or placebo, and the other nine RCTs assessed the effects of ginseng compared with placebo or no treatment on various conditions. The methodological caveats of the included trials make their contribution to the current clinical evidence of ginseng somewhat limited. However, the 20 newly added trials (66.7% of the 30 trials) may provide useful information for future trials. Ginseng appears to be generally safe, and no serious adverse effects have been reported.

**Conclusions:**

The clinical effects of ginseng have been tested in a wide range of conditions in Korea. Although the quality of RCTs published in the Korean literature was generally poor, this review is useful for researchers to access studies that were originally published in languages that they would otherwise be unable to read and due to the paucity of evidence on this subject.

## Introduction

Ginseng has a long history of medicinal use. In Korea, various processing methods have been developed to increase the efficacy and widen the clinical applicability of ginseng. Cultivated ginseng is classified into three types, depending on how it is processed: fresh ginseng (less than 4 years old), white ginseng (4–6 years old and dried after peeling), and red ginseng (harvested at 6 years old, steamed and dried) [1]. Red ginseng is not skinned before it is steamed or otherwise heated and subsequently dried. During the steaming process, ginseng starch is gelatinised, causing an increase in saponin content.

Ginseng is one of the most popular and best-selling herbal medicines worldwide [2]. Ginseng has been used as a medicine and as a health food by healthy and ill individuals around the world, especially in Asian countries [3]. Many clinical studies on ginseng have been performed to characterise its therapeutic properties, which include improving physical performance [4], sexual function [1], treating cancer [5], diabetes [6] and hypertension [7].

Currently, 13 systematic reviews of ginseng used to treat many conditions are available [1], [3], [6], [8]–[16]. Five of them include studies published in English only [10]–[12], [16], two analysed the results from English and Chinese databases [8], [9], and the other five searched several available databases, including Korean, Chinese, English and Japanese databases [1], [3], [6], [13], [14]. However, these reviews failed to include several recent studies published in Korea, where the name panax ginseng is from. Moreover, many Korean studies are not included in Western databases, and many Korean clinical trials on ginseng have only been reported in Korean journals. Therefore, it is necessary to summarise all of the available randomised clinical trials (RCTs) in the Korean literature to inform future systematic approaches to the study of ginseng. This summary could be valuable in providing ginseng research data that are not accessible by non-Korean researchers. The objective of this systematic review was to summarise RCTs assessing the efficacy and safety of ginseng that have been published in the Korean literature.

## Methods

### Data Sources

The following eight electronic Korean medical databases were searched without restriction of language from their respective inceptions up to December 2012 (Appendix S1): the Korean Studies Information Service System (KISS), DBPIA, Korea Institute of Science and Technology Information, Research Information Service System (RISS), Korea Med, Korean Medical Database (KM base), Oriental Medicine Advanced Searching Integrated System (OASIS) and the National Assembly Library. The search terms used were “ginseng”, “clinical” and Korean language terms related to ginseng and clinical trials. In addition, our own files and The Journal of Ginseng Research (http://www.ginsengres.org/main/) up to December 2012 were searched manually. The references in all located articles were also searched. Hard copies of all articles were obtained and read in full.

### Types of Studies

This review included parallel and cross-over RCTs that assessed the efficacy and safety of ginseng treatment. We excluded case studies, case series, uncontrolled trials and non-randomised clinical trials. Trials that failed to provide detailed results or in which ginseng was used in conjunction with conventional treatment were also excluded. Trials published in the form of dissertations and abstracts were included.

### Types of Participants

All articles describing an RCT on healthy people or patients with various disease conditions were included.

### Types of Interventions

Trials that included extracts of Korean ginseng or American ginseng or a commercial product made from Korean ginseng or American ginseng, regardless of age or dose, were included. According to the processing status, we also included fresh ginseng, white ginseng and red ginseng. We compared placebo or no treatment to ginseng therapies used alone or in combination with other conventional treatments.

### Data Extraction, Risk of Bias Assessment and Analysis

All articles were read by three independent reviewers (JC, TYC and THK) who extracted data from the articles according to predefined criteria: author information, total sample size, condition, age of the participants, intervention and control groups, dose per day, measure, main results, adverse events and language.

The risk of bias was assessed using the ‘Risk of Bias’ assessment tool from the Cochrane Handbook for Systematic Reviews of Interventions [17]. The following characteristics were assessed: sequence generation, allocation concealment, patient and personnel blinding, assessor blinding, reporting drop-out or withdrawal, intention-to-treat analysis and selective outcome report. If the article mentioned the protocol, the protocol was evaluated to determine whether all of the outcomes described in the protocol were reported in the original literature to evaluate selective outcome reporting. Our review used low (L), unclear (U) and high (H) as keys for judgments. Differences in opinions between the reviewers were settled through discussion.

Estimated effect size for each outcome of included studies was calculated comparing with each control intervention individually. Dichotomous data were presented as relative risk (RR) and continuous outcomes as mean difference (MD) with 95% CI (Confidence Interval). Analysis was conducted with Review manager 5.1 (Copenhagen: The Nordic Cochrane Centre, The Cochrane Collaboration, 2011).

## Results

### Study Selection and Description

**Figure 1:**
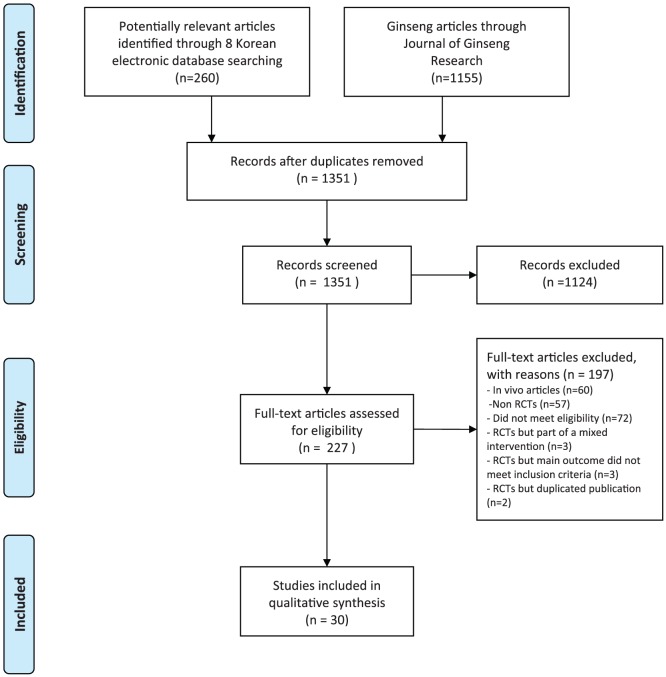
Flowchart of publication selection process. RCT: Randomized controlled trials.

We considered 1351 potentially relevant articles (Figure 1). After screening the abstracts and titles, we excluded 1124 studies. A total of 227 articles were read in full and evaluated. Subsequently, 197 were excluded because they described in vivo studies (60 articles), were non-randomised trials (57 articles), did not meet the eligibility criteria (72 articles), or were excluded for other reasons (8 articles). The eight RCTs excluded for other reasons were as follows: three studies employed a mixed intervention that included ginseng in the treatment group, including trials of HT008-1 (including ginseng, *Acanthopanax senticosus, Angelica sinensis* and *Scutellaria baicalensis*) [18], a mixture of ginseng plus *Paeoniae radix* [19] and interaction between ginseng and warfarin [20]. Five other studies [21]–[25] were excluded because they evaluated only serological outcomes [21]–[23] and duplicated publication [24], [25]. Finally, 30 RCTs met our inclusion criteria [26]–[55] (Tables 1 and 2). Twenty-eight trials originated from Korea [28]–[55], and two trials were conducted in Thailand [26] and the United Kingdom [27]. Sixteen studies [28], [32]–[41], [44]–[47], [53] were published in Korean, and 14 trials [26], [27], [29]–[31], [42], [43], [48]–[52], [54], [55] were written in English.

**Table 1:** Summary of randomised clinical studies of ginseng for healthy persons in Korean literatures.

First author (year)	Total sample size/Age(mean or range)	Intervention(n)	Dose per day (duration, forms)	Main outcomes	Main results(effect estimates)	Adverse events	Language
Pipat(1995) [26]	20/(A) 22.1±0.5; (B) 21.0±0.2	(A) Ginseng (10)	600 mg(8 wk,Capsule)	1) Maximum oxygen consumption	1) MD, 13.30[11.14, 15.46],P<0.0001	n.r.	English
		(B) Placebo (10)		2) V_E_/V_O2_ ratio	2) MD, 15.10[11.04, 19.16],P<0.0001		
				3) Anaerobic power	3) MD, 9.50[8.14, 10.86],P<0.0001		
				4) Anaerobic capacity	4) MD, 2.80[1.68, 3.92],P<0.0001		
				5) Leg muscle strength	5) MD, 24.60[20.43, 28.77],P<0.0001		
Kennedy(2007) [27]	16/38.3±10.3	(A) Ginseng(n.r.)	400 mg(20 wk,Capsule)	1) Cognitive measure	NS*	n.r.	English
		(B) Placebo (n.r.)		2) Mood and QOL			
				3) WHO-QOL questionnaire			
				4) Glucose-Regulatory parameters			
Kang(2009) [28]	39/(A) 27.5±5.1;(B) 25.6±3.8	(A) RG (21)	3000 mg(3 wk,Capsule)	Somatization (SCL-90R)	MD, n.r., P = 0.04	n.r.	Korean
		(B) Placebo (18)					
Kim(2009) [29]	23/(A) 37.6±4.6;(B) 38.2±4.3	(A) RG (11)	6000 mg(12 wk,Capsule)	QOL and Sexual function		n.r.	English
		(B) Placebo (12)		1) FSFI total score (%)	1) MD,−14.85[−29.80, 0.10],P = 0.02		
				2) SF-36 physical (%)	2) MD, 6.31[−11.11, 23.73],P = 0.48		
				3) SF-36 mental (%)	3) MD, −4.69[−22.74, 13.36],P = 0.61		
Lee(2010) [30]	15/(A) 23.5±1.2;(B) 23.4±2.17	(A) RG (8)	4500 mg (2 wk, n. r.)	Polysomnographic Variables on sleep		n.r.	English
		(B) Placebo (7)		1) Total sleep time (min)	1) MD, −54.40[−95.07, −13.73],P = 0.009		
				2) Sleep efficiency (%)	2) MD, −0.30[−11.18, 10.58], P = 0.96		
				3) Apnea hypopnea index (hour)	3) MD, 0.00[−4.99, 4.99], P = 1.00		
Yeo(2012) [31]	15/(A)19–25;(B) n.r.	(A) RG (8)	4500 mg (2 wk, Capsule)	Neurocognitive function test (Key items of the Vienna test system version IX)		n.r.	English
		(B) Placebo (7)		1) Median reaction time (msec)	1) MD, −59.40[−151.92, 33.12],P = 0.21		
				2) Wrong decision	2) MD, −0.12[−0.66, 0.42],P = 0.067		
				3) Right reaction	3) MD, 0.05[−0.34, 0.44],P = 0.80		
				4) No. of correct	4) MD, −5.20[−17.79, 7.39],P = 0.42		
				5) No. of incorrect	5) MD, 0.42[−1.69, 2.53],P = 0.70		
Seo(2004) [32]	160/22.4.±2.0	(A) RG (32)	3000 mg (4 wk, Capsule)		(A) vs (B) :	n.r.	Korean
		(B) Placebo (32)		1) Systolic blood pressure	1) MD, 4.69 [−2.52, 11.89],P = 0.20		
		*(C) WG (32)*		2) Diastolic blood pressure	2) MD, 3.44 [−1.67, 8.54],P = 0.18		
		*(D) AG (4years, 32)*		3) Pulse Rate	3) MD, −3.50 [−8.07, 1.07],P = 0.13		
		*(E) AG (6years, 32)*			*(A)+(C) vs (D)+(E):*		
					*1) MD, 0.31 [*−*14.88, 15.51],P = 0.97*		
					*2) MD, 3.62 [*−*8.20, 15.45.74],P = 0.55*		
					*3) MD,* −*3.57 [*−*12.26, 5.11],P = 0.42*		
				General Symptom Questionnaire	(A) vs (B) :		
				1) Headache	1) RR, 2.00[0.67, 5.98],P = 0.2150		
				2) Flushing	2) RR, 1.23[0.71, 2.12], P = 0.4541		
				3) Epistaxis	3) RR, 5.00[0.62, 40.44], P = 0.1313		
					*(A)+(C) vs (D)+(E):*		
					*1) RR, 2.00[0.86, 4.63]*		
					*2) RR, 1.1 [0.73, 1.76]*		
					*3) RR, 4.00[0.88, 18.11]*		
Yoon(2008) [33]	17/(A) 19.9±1.9	(A) RG (10)	3000 mg (12 wk, Pouch)	1) Maximum oxygen consumption	1) MD, −5.50 [−11.16, 0.16],P = 0.06	n.r.	Korean
	(B) 19.7±1.6	(B) Placebo+ET (7)		2) V_E_/V_O2_ ratio	2) MD, −3.10 [−5.33, −0.87],P = 0.006		
Jeong(2006) [34]	10/26.0±1.8	(A) Ginseng (n.r.)/RG (n.r.)/FRG (n.r.)	500 mg (2 wk, Capsule)	Cerebrovascular reactivity	P = 0.009†	none	Korean
		(B) Placebo (n.r.)					

ET: Endurance training; FSFI: Female sexual function index; FG: Fermented red ginseng, n.r.: not reported; NS: not significant; QOL: Quality of life; RG: Red ginseng; SF-36∶36 Item short-form health survey; SCR-90R: Symptomchecklist-90-revised score WG: White Ginseng.

* No numerical data available for calculating effect size. We added the results on the base of authors’ results.

† The original authors reported statistical significance but our calculation failed to do so.

Italic: The main outcome tested the effects of Korean ginseng compared with American ginseng.

**Table 2:** Summary of randomised clinical studies of ginseng for various conditions in Korean literatures.

First author (year)	Total sample size/Condition/Age(mean or range)	Intervention(n)	Dose per day(duration, forms)	Main outcomes	Mains results(effect estimates)	Adverse events	Language
Hong(2001) [35]	45/ED/54	(A) Ginseng (22)	900 mg	1) GEQ	1) RR, 3.00 [1.60, 5.64], P = 0.0006	n.r.	Korean
		(B) Placebo (23)	(16 wk, Powder)	2) Total IIEF	2) MD, 7.20 [-2.25, 16.65], P = 0.14		Dissertation
Choi(1999) [36]	64/ED/n.r.	(A) RG (37)	1800 mg	Questionnaire evaluation(not validated)		Constipation (A:2),	Korean
		(B) Placebo (27)	(12 wk, Capsule)	1) Libido	1) RR, 2.43 [1.13, 5.23], P = 0.0229	Gastric upsets (A:2, B:3)	
				2) Erection	2) RR, 2.19 [1.24, 3.86], P = 0.0069		
				3) Ejaculation	3) RR, 2.34 [0.98, 5.59], P = 0.0569		
				4) Sexual activity	4) RR, 1.75 [1.01, 3.02], P = 0.0443		
				5) Satisfaction	5) RR, 2.10 [1.11, 3.95], P = 0.0218		
Choi(2001) [37]	47/ED/(A)46.1±7.6	(A) RG (24)	1800 mg	1) GEQ	1) RR, 2.24 [1.04, 4.81], P = 0.0396	Gastric upsets (A:1, B:1)	Korean
	(B)45.4±8.9	(B) Placebo (23)	(8 wk, Capsule)	2) IIEF-Penetration	2) MD, 1.25 [1.16, 1.34], P<0.00001		
				3) IIEF-Maintained erection	3) MD,1.52 [1.44, 1.60], P<0.00001		
Ham(2009) [38]	69/ED/(A) 53.2±9.7	(A) RG (35)	800 mg (8 wk,Powder)	IIEF		Acute nasopharygitis (B:3)	Korean
	(B) 50.8±8.0	(B) Placebo (34)		1) Erectile function	1) MD, 3.60 [−0.09, 7.29], P = 0.06	Rhinitis (A:1)	
				2) Intercourse Satisfaction	2) MD, 1.10 [−0.34, 2.54], P = 0.13	Eczema (A:1)	
				3) Orgasmic Function	3) MD, 0.60 [−0.70, 1.90], P = 0.37	Skin disease (A:1)	
				4) Sexual desire	4) MD, 1.10 [0.30, 1.90], P = 0.007	Diarrhea (A:1)	
				5) Overall satisfaction	5) MD, 0.80 [−0.24, 1.84], P = 0.13	Anal bleeding (B:1)	
						Voice disorders(A:1)	
						Ophthalamalgia(A:1)	
						Perineal pain (A:1)	
						Chest pain (A:1)	
						Renal steone (n = 1)	
Kim(2006) [39]	35/ED/(A)43.6±14.1	(A) RG (23)	n.r. (12 wk,Capsule)	IIEF-5	MD, 0.30 [−2.44, 3.04], P = 0.83	Dyspepsia (B:1)	Korean
	(B)36.1±5.6	(B) Placebo (12)					
Kim(1999) [40]	21/Vasculogenic impotent/(A) 45.6	(A) RG (11)	2700 mg (12 wk, Capsule)	Total watts sexual functioning questionnaires	MD, 3.30 [−1.60, 8.20], P = 0.19	n.r.	Korean
	(B) 44.8	(B) Placebo (10)					
Yoo(1995) [41]	39/Gastric cancer/(A) 53.9±11.77	(A) Ginseng (20)	5400 mg (24 mth, Capsule)	NSIF		n.r.	Korean
	(B) 54.7±1.09	(B) No treatment (19)		1) Triceps skinfold thickness	1) MD, 0.70 [−4.48, 5.88], P = 0.79		
				2) Serum protein (mg/dL)	2) MD, 0.00 [−0.38, 0.38], P = 1.00		
				3) Serum albumin (mg/dL)	3) MD, 0.10 [−0.13, 0.33], P = 0.40		
				4) Serum transferrin (mg/dL)	4) MD, 32.00 [−7.21, 71.21], P = 0.11		
				5) Prognostic nutritional index	5) MD, 11.88 [−36.28, 60.04], P = 0.32		
				6) Blood lymphocyte (%)	6) MD, 3.20 [−3.10, 9.50], P = 0.63		
Suh(2007) [42]	47/Advanced colon cancer/(A) 65.6±8.9	(A) RG (23)	3000 mg (12 wk, Pouch)	Immune modulator following a curative surgery		none	English
	(B) 63.7±10.2	(B) No treatment (24)		1) IL-2(pg/ml)	1) MD, 4.30 [4.29, 4.31], P<0.00001		
				2) IL-8(pg/ml)	2) MD, −3.10 [−3.11, −3.09], P = 0.233		
				3)IL-10(pg/ml)	3) MD, −3.41 [−8.94, 2.12], P = 0.002		
Suh(2004) [43]	50/Advanced gastric cancer/(A) 55;(B) 56	(A) RG (24)	3000 mg (12 wk, Capsule)	Analysis of circulating interleukin		n.r.	English
		(B) No treatment (26)		1) IL-2	1) NS*		
				2) IL-10	2) NS*		
Suh(1998) [44]	72/Gastrointestinal carcinoma/(A)56.5±12.7	(A) RG plus (B) (32)	4500 mg (18 months, Capsule)	Postoperative Immune response		n.r.	Korean
	(B) 54.6±13.37	(B) Chemotheapy and immunotherapy (40)		1) Total leukocyte counts	1) MD, 2.74 [−0.53, 6.01], P = 0.10		
				2) Suppresoor/cytotoxic cell counts	2) MD, 177.00 [65.14, 288.86], P = 0.002		
				3) Helper/inducer cell counts	3) MD, 184.80 [1.73, 367.87], P = 0.05		
				4) Natural killer cell	4) MD, 180.00 [4.43, 355.57], P = 0.04		
Kim(2007) [45]	70/Chronic gastritis/(A) 48.0±0.5	(A) RG (36)	2700 mg (10 wk, Capsule)	1) *H.pylori* eradication rate	1) RR, 1.15 [0.95, 1.41], P = 0.1543	n.r.	Korean
	(B) 52.4±0.5	(B) Placebo (34)		2) 8-OHdG attenuation	2) NS†		
Choi(1997) [46]	31/NIDDM/(A) 51.6±10.9	(A) RG (14)	2700 mg (24 wk, Capsule)	1) FBS(mg/dl)	1) MD, −25.10 [−53.71, 3.51], P = 0.09	n.r.	Korean
	(B) 50.4±7.5	(B) No treatment (17)		2) PP2hrBG(mg/dl)	2) MD, 27.10 [−29.58, 83.78], P = 0.35		
Kim(2008) [47]	38/Type 2 DM/(A) 56.00±7.17	(A) RG (20)	780 mg (12 wk, Capsule)	1) FPG (mmol/L)	1) MD, −0131 [–1125, 0163], P = 0.152	n.r.	Korean
	(B) 51.22±10.77	(B) Placebo (18)		2) FPI (pmol/L)	2) MD, −0118 [–0182, 0146], P = 0.158		Dissertation
				3) HbA1c (%)	3) MD, −0121 [–0194, 0152], P = 0.157		
				4) HOMA IR (mmol/L)	4) MD, −0134 [–0198, 0130], P = 0.130		
Kim(2009) [48]	32/Androgenic alopecia/(A) 37.5	(A) RG (17)	3000 mg(24 wk, Capsule)	1) Hair density (n/cm2)	1) MD, 15.57 [−5.98, 37.12], P = 0.15(12weeks); MD, 21.23 [1.10, 41.36], P = 0.04(24weeks)	Dyspepsia (A:1)	English
	(B) 43.3	(B) Placebo (15)		2) Hair thickness (mm)	2) MD, 0.01 [−0.00, 0.02], P = 0.15(12weeks); MD, 0.01 [−0.00, 0.02], P = 0.27 (24weeks)		
				3) Patient satisfaction by questionnaire(not validated)	3) RR, 1.39 [0.73, 2.64], P = 0.320		
Chung(2010) [49]	20/Coronary artery diseases/62.4±3.1	(A) RG (n.r.)	2700 mg (20 wk, n.r.)	1) Systolic blood pressure	1) NS*	n.r.	English
		(B) Placebo (n.r.)		2) Vascular stiffness	2) NS*		
Park(2010) [50]	90/Dry mouth/(A) 45.9±16.1; (B) 48.3±15.8	(A) RG (44)	6000 mg (10 wk, Capsule)	1)VAS	1) MD, −0.08 [−1.02, 0.86], P = 0.347; Subgroup: MD, −0.27 [−1.27, 0.73](in women, 40 yr–59 yr), P = 0.228	Dyspepsia (A:3, B:2),diarrhea (A:1, B:3), itching sensation (A:1, B:2), mild fever (A:1, B:1), palmar sweating (A:1), sleep disturbance (B:1)	English
		(B) Placebo (46)		2) Dry mouth-related symptom questionnaire	2) MD, −1.10 [−5.43, 3.23], P = 0.073		
Kim(2010) [51]	36/Glaucoma/59.0±12.5	(A)RG (18)	4500 mg (12 wk, Capsule)	Visual ocular blood flow	NS*	n.r.	English
		(B) Placebo (18)					
Kwon(2011) [52]	50/Obese/(A) 40.9±9.7	(A) RG (22)	6000 mg(8 wk, Capsule)	1) Weight Loss	1) RR, 5.22 [0.26, 102.93],P = 0.2776	None	English
	(B) 46.5±9.8	(B) Placebo (23)		2) BMI	2) NS†		
				3) KOQOL	3) NS†		
				4) HDL	4) NS†		
				5) T-Chol	5) NS†		
Park(2012) [53]	48/Metabolic syndrome/(A) 43.1±10.6	(A) RG (23)	4500 mg(12 wk, Powder)	1) SBP	1) MD, 5.00[−8.12, 18.12], P = 0.46	n.r.	Korean
	(B) 46.2±11.0	(B) Placebo (25)		2) DBP	2) MD, 2.50[−6.63, 11.63], P = 0.59		
				3) T-Chol	3) MD, 1.70 [−57.42, 31.57], P = 0.91		
				4) Oxidized LDL	4) MD, −11.10[−57.42, 35.22], P = 0.64		
Lee(2010) [54]	76/dyspepsia and indigestion/51.6±14.5	(A) RGpuls (B) (31)	2700 mg (10 wk, Capsule)	*H.pylori* eradication rates	RR, 1.34 [0.98, 1.83], P = 0.0677	n.r.	English
		(B) Eradication regimen (45)					
Lee(2007) [55]	82/Alzheimer/66.1±9.1	(A) Ginseng (50)	4500 mg (12 wk, Powder)	Cognitive improvement		none	English
		(B) No treatment (32)		1) ADAS score	1) MD, −2.85 [−5.40, −0.30], P = 0.03		
				2) MMSE score	2) MD, 1.83 [0.50, 3.16], P = 0.007		

ADAS score: Alzheimer’s disease assessment scale; BMI: body mass index; DBP: diastolic blood pressure; DM: diabetes mellitus; ED: erectile dysfunction; FBS: fasting blood glucose; GEQ: global efficacy question; HDL: high density lipoprotein; IIEF: International index of erectile function; KOQOL: Korean version of obesity-related quality of life; MMSE score: Mini-mental status exam; n.r.: not reported; NS: not significant; NSIF: nutritional status and immune function; NIDDM: non-insulin dependent diabetes mellitus; PP2hrBG: Postprandial 2hour blood glucose; RG: red ginseng; SBP: systolic blood pressure; SOD: superoxide dismutase; T-Chol: total cholesterol; VAS: visual analog scale; 8-OHdG: 8-hydroxydeoxyguanosine.

* No numerical data available for calculating effect size. We added the results on the base of authors’ results.

† The original authors reported statistical significance but our calculation failed to do so.

Italic: The main outcome tested the effects of Korean ginseng compared with American ginseng.

Of the 30 trials that met our inclusion criteria, two dissertations were included [35], [47]. The key data from studies in healthy persons are summarised in Table 1 [26]–[34], and the data regarding other various conditions are summarised in Table 2 [35]–[55]. The included RCTs used ginseng powder either in raw (4 studies) [35], [38], [53], [55] or in capsules (22 studies) [26]–[29], [31], [32], [34], [36], [37], [39]–[41], [43]–[48], [50]–[52], [54] and extract preparation (2 studies) [33], [42], while two studies [30], [49] did not report the preparation type of ginseng. They addressed a wide range of conditions: generally healthy (i.e., studies in healthy individuals) [26]–[34], erectile dysfunction [35]–[40], gastric cancer [41], [43], colon cancer [42], gastrointestinal carcinoma [44], chronic gastritis [45], diabetes mellitus [46], [47], androgenic alopecia [48], coronary artery [49], dry mouth [50], glaucoma [51], obesity [52], metabolic syndrome [53], dyspepsia and indigestion [54] and Alzheimer’s disease [55].

### Risk of Bias in the Included Studies

The risk of bias of the trials was variable (Table 3). Nine studies [28], [32], [34], [44], [47], [50]–[53] used methods of random sequence generation. Of those, four RCTs [28], [47], [51], [53] used a computerised randomisation method. However, the risk of bias in the sequence generation was high in five RCTs [32], [34], [44], [50], [52]. Most studies did not clearly report how the allocation concealment was generated; only five RCTs [27], [32], [50]–[52] employed allocation concealment. Seventeen RCTs [26]–[29], [31], [32], [34], [35], [38], [40], [47]–[53] reported double blinding (patient/practitioner). Only three of these RCTs [32], [34], [35] adopted assessor blinding. Forteen RCTs [27], [29], [31], [33], [36], [37], [40], [43], [45], [46], [48], [52], [53], [55] had a high risk of drop-out or withdrawal bias. Only six RCTs [26], [32], [35], [41], [47], [51] used intention-to-treat analysis (ITT). The risk of bias in selective outcome reporting was low in seven RCTs [26], [32], [45], [50], [51], [53], [54], high in two studies [32], [52] and unclear in the remaining studies. There were no difference between low risk of bias and high risk of bias when comparing the effectiveness of ginseng.

**Table 3:** Risk of bias* for included randomized clinical trials.

Study	Random sequence generation	Allocation concealment	Blinding		Reporting drop out or withdrawal	incomplete outcome data	Selective outcome reporting
			Patient and personnel	Assessor			
Pipat(1995) [26]	U	U	L	U	L	L	L
Kennedy (2007) [27]	U	L	L	U	H	U	U
Kang(2009) [28]	L	U	L	U	L	H	U
Kim(2009) [29]	U	U	L	U	H	H	U
Lee(2010) [30]	U	U	H	U	L	U	U
Yeo(2012) [31]	U	U	L	U	H	H	U
Seo(2004) [32]	H	L	L	L	L	L	H
Yoon(2008) [33]	U	U	U	U	H	H	U
Jeong(2006) [34]	H	U	L	L	U	U	U
Hong(2001) [35]	U	U	L	L	L	L	U
Choi(1999) [36]	U	U	H	H	H	H	U
Choi(2001) [37]	U	U	U	U	H	H	U
Ham(2009) [38]	U	U	L	U	L	H	U
Kim(2006) [39]	U	U	U	U	U	U	U
Kim(1999) [40]	U	U	L	U	H	H	U
Yoo(1995) [41]	U	U	H	H	L	L	U
Suh(2007) [42]	U	U	H	H	U	H	U
Suh(2004) [43]	U	U	H	H	H	H	U
Suh(1998) [44]	H	H	H	H	U	U	U
Kim(2007) [45]	U	U	H	U	H	H	L
Choi(1997) [46]	U	U	H	H	H	H	U
Kim(2008) [47]	L	U	L	U	L	L	U
Kim(2009) [48]	U	U	L	U	H	H	U
Chung(2010) [49]	U	U	L	U	L	H	U
Park(2010) [50]	H	L	L	U	L	H	L
Kim(2010) [51]	L	L	L	U	L	L	L
Kwon(2011) [52]	H	L	L	U	H	H	H
Park(2012) [53]	L	U	L	U	H	H	L
Lee(2010) [54]	U	U	H	H	L	H	L
Lee(2007) [55]	U	U	H	H	H	H	U

* Domains of quality assessment based on Cochrane tools for assessing risk of bias.

L, low risk of bias; H, high risk of bias; U, unclear risk of bias.

### Healthy Persons

#### Ginseng versus placebo

**Two RCTs** [**26**], [**27**] **evaluated the effects of ginseng compared with a placebo on exercise capacity and cognitive performance in healthy individuals.** One RCT [26] compared the effects of ginseng with placebo on exercise capacity and showed a significant effect for increasing the maximum oxygen consumption, anaerobic variables and leg muscle strength. The other RCT [27] reported the superiority of ginseng over placebo for treating mood, quality of life and memory performance. However, no significant difference was found between ginseng and placebo.

#### Red ginseng versus placebo

Six RCTs [28]–[33] assessed the effects of red ginseng compared with placebo. One RCT showed significant effects of red ginseng on somatic symptoms [28]. Second RCT showed beneficial effects of red ginseng on sexual function [29]. Third RCT reported significant effects on total sleep time [30]. Forth RCT [32] compared the effects of red ginseng with placebo and failed to show any change in blood pressure, pulse rate or body temperature. The authors reported the effects of ginseng on general health symptom from same trial and also failed to beneficial effects of ginseng on general health symptoms in [25]. Fifth RCT also failed to show significant effects on cognitive function [31]. Sixth RCT reported significant effects of red ginseng on anaerobic performance [33].

#### Ginseng, red ginseng and fermented red ginseng versus placebo

One RCT [34] evaluated the effects of ginseng, red ginseng, and fermented red ginseng on cerebral hemodynamics compared with placebo. However, no group showed significant differences from the placebo.

#### Erectile dysfunction

Six RCTs [35]–[40] tested the effects of ginseng or red ginseng compared with placebo on sexual function using questionnaires. Four studies reported positive effects of ginseng on at least one outcomes related with erectile function [35]–[38], while the other two RCTs failed to do so [39], [40].

### Gastric and Colon Cancer

Four RCTs [41]–[44] tested the effects of ginseng or red ginseng compared with no treatment on nutritional status [41], immune function [42]–[44]. Of these, one RCT [42] reported significantly improved immune modulation after surgery. The remaining RCTs [41], [43], [44] failed to show a significant effect of ginseng on immune response, or nutritional status.

### Diabetes Mellitus

Two RCTs [46], [47] compared the effects of red ginseng on diabetes mellitus with no treatment or placebo. One RCT [46] did not show any superior effect of red ginseng on FBS (fasting blood glucose) or PP2H (postprandial 2 hour). The other RCT [47] also indicated no favourable effects of red ginseng on FPG, FPI or HbA1c.

### Other Conditions

Nine RCTs assessed the effects of ginseng or red ginseng compared with a placebo or no treatment on androgenic alopecia [48], coronary artery [49], chronic gastritis [45], dry mouth [50], dyspepsia and indigestion [54], glaucoma [51], obesity [52], metabolic syndrome [53] and Alzheimer’s disease [55]. In most of the studies mentioned above, no significant difference was found between red ginseng and placebo. However, one of these RCTs reported beneficial effects of ginseng on the Korean version of obesity-related quality of life (KOQOL) scores in obese patients and MMSE (mini-mental status exam) and ADAS (Alzheimer’s disease assessment scale) scores after 12 weeks [55]. One RCT [54] showed that red ginseng increased the *H. pylori* eradication rate in dyspepsia and indigestion, while the other RCT failed to do so in chronic gastritis [45].

### Adverse Events

Six RCTs [36]–[39], [48], [50] mentioned adverse events, while the other RCTs did not. None of the RCTs reported any serious adverse effects. Two RCTs [36], [37] reported gastric upset in two groups, and one of these [36] also reported constipation in the intervention group. The other RCTs [38], [39], [48], [50] reported various adverse symptoms that were not closely related to the intervention. Of these RCTs, one RCT [50] had various adverse effects and the largest dose of red ginseng of the included trials.

## Discussion

This review represents a systematic assessment of RCTs that are related to the effectiveness of ginseng published in the Korean literature. The clinical effects of ginseng have been tested for a wide range of conditions in Korea. Most RCTs published in the Korean literature have not been included in up-to-date systematic reviews. Our review aimed to summarise all the RCTs on ginseng in the Korean literature regardless of treated conditions. Nine trials included in this review reported results on exercise capacity, cognitive performance, somatic symptoms, quality of life, and sleeping in healthy persons [26]–[34]. Twenty-one RCTs tested ginseng or red ginseng compared with placebo or no treatment in erectile dysfunction, gastric and colon cancer, diabetes mellitus and other conditions [35]–[55]. This review may serve as a foundation for future systematic reviews and further studies, but the small sample size provides limited contribution. Compared to previous reviews [1], [3], [6], [8]–[16], we identified 20 new RCTs [26]–[31], [33], [38], [39], [41]–[45], [48], [49], [52]–[55] and successfully updated the information for therapy. Our ginseng review provides people the opportunity to access studies that were originally published in languages that they would otherwise be unable to read.

It is meaningful to compare discrepancies or agreements for each condition in the included studies with the conclusions of previous systematic reviews (Table 4). We updated our review with 20 new RCTs (66.7% of the 30 trials) on healthy people (7 trials) [26]–[31], [33], erectile dysfunction (2 trials) [38], [39], gastric and colon cancer (5 trials) [41]–[45], androgenic alopecia (1 trial) [48], coronary artery (1 trial) [49], obesity (1 trial) [52], metabolic syndrome (1 trial) [53], dyspepsia and indigestion (1 trial) [54], and Alzheimer’s disease (1 trial) [55]. One systematic review [1] suggested the effects of red ginseng on erectile dysfunction with 7 included RCTs. We found 2 additional Korean RCTs [38], [39] that showed positive effects of ginseng on ED. These studies can add more favourable effects of ginseng for this condition. One RCT [55], which assessed the effects of ginseng on Alzheimer’s disease, reported positive results on improving symptoms. However, this study was already included in a previous systematic review as a duplicate publication, and it could not contribute to the current evidence.

**Table 4:** Comparison between RCTs already included in the SR and eligible Korean RCTs.

First author(year)	Condition	Type of database	Number of primary studies (Korean)	Author’s conclusion (quote)	SR’s result (+/−)	Newly Eligible Korean RCTs compared with previous SRs	
						Number of RCT [ref]	Result(+/−)
Jang(2008) [1]	Erectile dysfunction	English, Korean, Chinese, Japanese	7(4)	… provide suggestive evidence	**+**	2[38], [39]	**+**
Kim(2011) [6]	Diabetes	English, Korean, Japanese	3(2)	…. effectiveness. is not convincing.	**–**	0	**–**
Jia(2012) [8]	Ischemic heart disease	English, Chinese	18(0)	… more effective than..further. to verify the efficacy.	**+**	0	**+**
An(2011) [9]	obstructive pulmonary	English, Chinese	12(0)	… show promising evidence … is uncertain due to potential risk of bias of.	**+**	0	**+**
Seida(2011) [10]	Common Cold	English	5(0)	. significantly reduced….insufficient evidence …	**+**	0	**+**
Seely(2008) [11]	Pregnancy and Lactation	English	Not clear	. conflicting evidence … no human studies.	**–**	Not clear	**–**
Buettner (2006) [12]	Cardiovascular	English	31(3)	.not support the use of. to treat cardiovascular risk factors.	**+/−**	0	**+/−**
Lee(2009) [13]	Alzheimer	English, Korean, Chinese	2(2)	The evidence…is scarce and inconclusive.	**+/−**	1[55]	**+**
Hur(2010) [14]	Hypertension	English, Korean, Chinese, Japanese	5(0)	show.significant. limited evidence …	**+/−**	0	
Lee(2011) [3]	various conditions	English, Korean, Chinese	57(9)	…finding a strong positive potential….	**+/−**	22[26]–[31], [33], [38], [39], [41]–[45], [48]–[55]	**+/−**
Vogler(1999) [15]	various conditions	English	16(0)	……efficacy.is not established beyond reasonable doubt….	**+/−**	30[26]–[55]	**+/−**
Shergis(2012) [16]	various conditions	English	65(2)	. promising results,….implications for several diseases….	**+/−**	28[26]–[49], [52]–[55]	**+/−**

RCT: randomized controlled trial; SR: systematic review; +: positive; −: negative; +/−: unclear.

One important question of these studies concerns the safety of ginseng. Ginseng appears to be generally safe, and no serious adverse effects have been reported. Adverse effects were noted in six of the RCTs included in this study [36]–[39], [48], [50]. None reported any serious adverse effects. However, the possibility of adverse effects caused by high doses of ginseng should be generally considered with caution. Therefore, another question is whether the therapeutic effects of ginseng depend on the form of ginseng and the amounts of various constituents in the preparation. Both the optimum dose and the ideal form of ginseng are currently unknown. No clinical trial comparing dosages or forms of ginseng has yet been published.

In the studies included in our analysis, the largest dose of ginseng used was 5.4 g [41], and the minimal dose of ginseng was 0.4 g daily [27]. The largest dose of red ginseng was 6.0 g [29], [50] daily, and the minimal dose of red ginseng was 0.8 g daily [38]. Hence, all of the RCTs in our review reported various doses of ginseng or red ginseng. Of the 30 RCTs included here, 22 RCTs used ginseng powder in capsules [26]–[29], [31], [32], [34], [36], [37], [39]–[41], [43]–[48], [50]–[52], [54], likely because ginseng capsules are more amenable to blinding than powder [35], [38], [53], [55] or extract preparations [33], [42]. It is clear that further clinical trials comparing the dosage or forms of ginseng are required.

Assuming that ginseng is a beneficial treatment for a wide range of conditions, its possible mechanisms of actions may be of interest. Like all herbal extracts, ginseng preparations are complex mixtures of multiple pharmacologically active ingredients. The most important and best researched of the active ingredients in ginseng are the ginsenosides, a diverse group of triterpenoidal saponins. Approximately 150 different ginsenosides have been identified to date. These compounds have complex biological activities. The mechanisms of action of ginseng are therefore diverse, complex and often somewhat unclear [56]. Further basic research is needed to fully understand the mechanisms of action of ginseng.

We also wish to highlight some of the difficulties inherent in research on ginseng and offer some suggestions for future research. First, researchers must use an appropriate random component for sequence generation, such as a computerised random number generator or coin toss. An appropriate randomised controlled trial design, more than any other factor, can have a powerful and immediate impact on patient care. However, appropriate randomisation was described in only four ginseng trials [28], [47], [51], [53] in the Korean literature. This may lead to selection bias and exaggerated treatment effects. Hence, more rigorous randomisation should be applied to future studies. Second, most of the included RCTs used a double-blinding procedure, but only three RCTs [32], [34], [35] used assessor blinding. Those that failed to do so are at risk of detection bias. That is, although all of the RCTs used a placebo or no treatment as a control group, none reported the success of blinding or the degree of unblinding due to the distinct taste and smell of ginseng. Therefore, the success of the blinding procedures should be assessed. Third, as noted, no clinical trial comparing dose dependency has yet been published. Studies with comparable controls would help to establish or contribute to the current evidence for the efficacy of ginseng.

This systematic review has several limitations. Although extensive efforts were made to retrieve all of the RCTs in the Korean literature, we cannot be certain that our searches located all relevant RCTs. In fact, the Korean database may have incorrectly reported some search results, and several early papers may be missing from the search.

In conclusion, ginseng is a popular herbal medicine that is used worldwide for a broad range of indications in the Korean literature. Although the quality of RCTs published in the Korean literature was generally poor, this review is useful for researchers to access studies that were originally published in languages they would otherwise be unable to read and due to the paucity of evidence on this subject. The results of this systematic review showed that ginseng appears to be effective for various medical conditions, particularly exercise capacity, somatic symptoms, erectile dysfunction, advanced colon cancer, diabetes, dyspepsia, indigestion and Alzheimer’s disease. However, the main limitation of our analysis was that nearly all the included trials were evaluated as having a high risk of bias and no difference between low risk of bias and high risk of bias when comparing the effectiveness of ginseng. As such, it is necessary to conduct further RCTs that are of high quality and with larger sample sizes to contribute to forming a definitive conclusion.

## Supporting Information

Appendix S1 The list of databases searched in this review.
(DOCX)

Checklist S1 PRISMA checklist
(DOC)
