# Supplementary material for: Ginseng for Health Care: A Systematic Review of Randomized Controlled Trials in Korean Literature
Source: PLoS One. 2013 Apr 1;8(4):e59978. doi: 10.1371/journal.pone.0059978 (PMC3613407; doi:10.1371/journal.pone.0059978)
Supplement: Appendix S1 — The list of databases searched in this review. (DOCX) [file pone.0059978.s001.docx]

**Appendix S1. The list of databases searched in this review**

| **Database Title** | **Publisher** | **Web site** |
| --- | --- | --- |
| Korean studies Information Service System (KISS) | Korean studies information | http://kiss.kstudy.com/ |
| DBPIA | Kyobo Book Center & Nurimedia (Korea) | http://www.dbpia.co.kr/ |
| Korea Institute of Science and Technology Information | Korea Institute of Science and Technology Information | http://society.kisti.re.kr/main.html |
| Research Information Service System (RISS) | Korea Education & Research Information Service | http://www.riss.kr/index.do |
| Korea Med | Korean Association of Medical Journal Edition | http://www.koreamed.org/SearchBasic.php |
| Korean Medical Database (KM base) | Medical Research Information Center (Korea) | http://kmbase.medric.or.kr/ |
| Oriental Medicine Advanced Searching Integrated System (OASIS) | Korea Institute of Oriental Medicine | http://oasis.kiom.re.kr/main.jsp |
| National Assembly Library | National Assembly Library of the Republic of Korea | [www.nanet.go.kr](http://www.nanet.go.kr) |
